# CHERISH (collaboration for hospitalised elders reducing the impact of stays in hospital): protocol for a multi-site improvement program to reduce geriatric syndromes in older inpatients

**DOI:** 10.1186/s12877-016-0399-7

**Published:** 2017-01-09

**Authors:** Alison M. Mudge, Merrilyn D. Banks, Adrian G. Barnett, Irene Blackberry, Nicholas Graves, Theresa Green, Gillian Harvey, Ruth E. Hubbard, Sharon K. Inouye, Sue Kurrle, Kwang Lim, Prue McRae, Nancye M. Peel, Jessica Suna, Adrienne M. Young

**Affiliations:** 1 Royal Brisbane and Women’s Hospital, Metro North Hospital and Health Service, Brisbane, Australia; 2 Institute of Health and Biomedical Innovation and School of Public Health and Social Work, Queensland University of Technology, Brisbane, Australia; 3 John Richards Initiative, Australian Institute of Primary Care and Ageing, La Trobe University, Albury-Wodonga, Australia; 4 School of Nursing, Queensland University of Technology, Brisbane, Australia; 5 University of Adelaide, Adelaide, Australia; 6 Centre for Research in Geriatric Medicine, Faculty of Medicine, University of Queensland, Brisbane, Australia; 7 Harvard Medical School, Beth Israel Deaconess Medical Centre, Institute for Aging Research, Hebrew Senior Life, Boston, USA; 8 Cognitive Decline Partnership Centre, Faculty of Medicine, University of Sydney, Sydney, Australia; 9 Melbourne Medical School, University of Melbourne, Melbourne, Australia

**Keywords:** Hospital care organisation, Elderly, Delirium, Functional decline, Falls, Pressure injuries, Incontinence, Implementation framework, Facilitation, Aged, Acute care, Hospitalisation, Pressure ulcer, Urinary incontinence, Implementation

## Abstract

**Background:**

Older inpatients are at risk of hospital-associated geriatric syndromes including delirium, functional decline, incontinence, falls and pressure injuries. These contribute to longer hospital stays, loss of independence, and death. Effective interventions to reduce geriatric syndromes remain poorly implemented due to their complexity, and require an organised approach to change care practices and systems. Eat Walk Engage is a complex multi-component intervention with structured implementation, which has shown reduced geriatric syndromes and length of stay in pilot studies at one hospital. This study will test effectiveness of implementing Eat Walk Engage using a multi-site cluster randomised trial to inform transferability of this intervention.

**Methods:**

A hybrid study design will evaluate the effectiveness and implementation strategy of Eat Walk Engage in a real-world setting. A multisite cluster randomised study will be conducted in 8 medical and surgical wards in 4 hospitals, with one ward in each site randomised to implement Eat Walk Engage (intervention) and one to continue usual care (control). Intervention wards will be supported to develop and implement locally tailored strategies to enhance early mobility, nutrition, and meaningful activities. Resources will include a trained, mentored facilitator, audit support, a trained healthcare assistant, and support by an expert facilitator team using the i-PARIHS implementation framework. Patient outcomes and process measures before and after intervention will be compared between intervention and control wards. Primary outcomes are any hospital-associated geriatric syndrome (delirium, functional decline, falls, pressure injuries, new incontinence) and length of stay. Secondary outcomes include discharge destination; 30-day mortality, function and quality of life; 6 month readmissions; and cost-effectiveness. Process measures including patient interviews, activity mapping and mealtime audits will inform interventions in each site and measure improvement progress. Factors influencing the trajectory of implementation success will be monitored on implementation wards.

**Discussion:**

Using a hybrid design and guided by an explicit implementation framework, the CHERISH study will establish the effectiveness, cost-effectiveness and transferability of a successful pilot program for improving care of older inpatients, and identify features that support successful implementation.

**Trial registration:**

ACTRN12615000879561 registered prospectively 21/8/2015.

## Background

People aged 65 and over account for more than half of hospital bed days [1], and their stay is commonly complicated by non-disease specific complications known as geriatric syndromes [2–5]. Delirium, functional decline, falls, incontinence and pressure injuries occur frequently in older inpatients, and represent the interaction between baseline vulnerability (conceptualised as frailty), acute illness or surgery and the hospital care environment [6–8]. Hospital-associated geriatric syndromes share risk factors including functional impairment, mobility impairment, cognitive impairment and malnutrition. They frequently co-exist, and are associated with longer hospitalisations, increased dependency and greater risk of institutional care and death [2, 3, 5, 8–10].

Integrated, multi-component interventions which address the common shared risk factors can significantly reduce the risk of geriatric syndromes and may reduce hospital length of stay [8, 11–13]. Components include “basic” strategies such as encouraging early mobilisation and functional independence, supporting nutrition and hydration, and providing reorientation and meaningful personal engagement. However, these strategies are not consistently applied within the complex acute care system [6, 14, 15]*,* with recognition of a large number of barriers at the level of the patient (e.g. symptoms, motivation), staff (e.g. knowledge, attitudes, time, skills, responsibility, leadership) and system (e.g. culture, resources, competing priorities) [16–23].

Eat Walk Engage [24] is a complex multi-component intervention designed to identify and address these barriers on non-geriatric wards. A complex intervention is one with multiple components, behaviours, groups, and/or outcomes impacted [25]. Eat Walk Engage aims to improve early mobility and functional independence, enhance oral nutritional intake, and improve opportunities for interactions and meaningful cognitive activities. The program uses the integrated Promoting Action on Research Implementation in Health Services (i-PARIHS) framework designed to support implementation of complex interventions [26]. A skilled facilitator assists the local multidisciplinary team (MDT) to prioritise areas for improvement, identify barriers and enablers, develop solutions tailored to the local context, and evaluate using iterative cycles of change and measurement [26]. In pilot studies, Eat Walk Engage decreased hospital-acquired geriatric syndromes and decreased length of stay [24, 27].

The Collaborative for Hospitalised Elders: Reducing the Impact of Stays in Hospital (CHERISH) study will be a hybrid evaluation of Eat Walk Engage, using outcome and process evaluation to study both intervention effectiveness, and the change processes involved in implementation [28]. The aim of the outcome evaluation is to evaluate the effectiveness and cost-effectiveness of the Eat Walk Engage program for inpatients aged 65 years and older receiving care on medical and surgical wards. The resources, interventions, goals and hypothesised outcomes of the program are outlined in Table 1. The main CHERISH study hypotheses are that, compared to control wards, wards implementing Eat Walk Engage will demonstrate:

- Reduction in hospital-related geriatric syndromes (delirium, functional decline, falls, pressure injuries and new incontinence)
- Reduction in length of stay under the treating team
- Reduction in institutional care (rehabilitation or other subacute care, hospital transfers, or new residential aged care placement).

**Table 1:** Eat Walk Engage program resources, intervention components, program goals, outcomes and evaluation methods. FTE full time equivalent. MDT existing multidisciplinary team

RESOURCES	INTERVENTION COMPONENTS	PROGRAM GOALS	OUTCOMES
Trained Eat Walk Engage facilitator (0.4 FTE per ward) Expert facilitation team Trained Eat Walk Engage assistant (0.5 FTE per ward)	Under guidance of the facilitator: • MDT develops shared understanding and improvement goals • MDT identifies local barriers and enablers for nutrition, mobility and cognitive engagement • MDT clarifies roles and opportunities for delegation to assistant • MDT initiates small cycle improvements with re-evaluation	Higher proportion of older patients achieve: • Early and adequate nutrition • Early mobility and independence • Meaningful activities and participation	Geriatric syndromes Length of stay Institutional discharge Health care costs
Program staff costs Training materials and time	i-PARIHS mapping Baseline interviews, audits MDT meeting minutes Facilitator journals Facilitator interviews MDT service patterns	Patient interviews Activity mapping Mealtime audits	Geriatric syndromes Length of stay Discharge destination 30 day health status Hospital readmissions

A cost-effectiveness evaluation will determine whether improvements in health benefits measured in quality adjusted life years are justified against the additional costs of implementing the program. The process evaluation will help to understand how and where the program worked, by testing the effect on care processes and outcomes (Table 1) and by exploring how key contextual features of different sites contributed to implementation success or failure.

## Methods

### Setting

The study will be conducted in four hospitals, including two metropolitan teaching hospitals and two large regional hospitals, in two Hospital and Health Services (HHS) serving populations in South-East Queensland, Australia. During study planning, senior clinical and administrative leaders from these hospitals committed resources to participate in this partnership project, and have assisted the study team to identify two medical or surgical wards in each hospital admitting at least 50% of admitted patients aged over 65, which are suitable for participation in the study. The 8 selected wards are three general medical wards, two specialty medicine wards, one orthopaedic ward and two general surgical wards.

### Study design

We will use a cluster randomised control design, randomising one ward within each hospital (cluster) to implement the Eat Walk Engage program (intervention) and the other to continue usual care (control). Allocation will be undertaken by the off-site study statistician based on computer-generated random numbers using R [29].

**Fig. 1:**
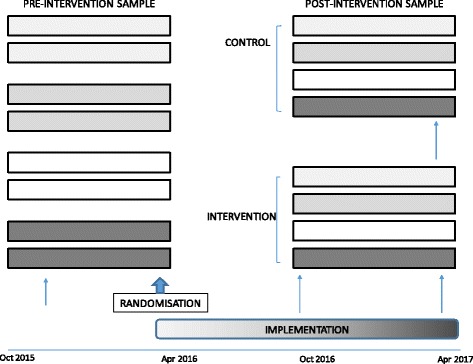
Study design. Pre-intervention data collected for 6 months on 8 wards (2 in each hospital, as indicated by shading colour) prior to randomisation. Eat Walk Engage implemented in 4 intervention wards. Post-intervention data collected on all wards 8 months after randomisation. Interview and audit data collection points are indicated by arrows

Data will be collected on all 8 wards before and after an intervention period, with additional process data collected during implementation on intervention wards (Fig. 1). Outcome evaluation will compare measures in pre-intervention and post-intervention samples on the intervention wards, compared to measures in samples from the same time on control wards. This study design allows measurement of changes in processes and outcomes following intervention (to understand mechanisms of any observed outcome improvement) while controlling for systematic changes at hospital level which may affect the outcomes of interest. Patient-level data collection is planned for October 2015-March 2016 (pre-intervention) and October 2016-March 2017 (post-intervention).

Study oversight will be provided by a Steering Committee consisting of the Chief Investigators which is responsible for the protocol, study timelines, and supervision of analysis by the data manager (JS) and statistician (AB). Implementation will be overseen by a working group including experienced implementation scientists (GH, IB), expert and site facilitators, site investigators and a consumer representative.

### Implementing Eat Walk Engage

Implementation will be guided by the i-PARIHS framework, designed to support implementation of complex interventions in healthcare [26]. The central element is enabling facilitation, recognising the importance of tailoring strategies to the local recipients (e.g. patient type, staff mix) and context (e.g. culture, resources, priorities). Each site will be provided with a facilitator (0.4 full time equivalent) who will be a health professional recruited within the study hospital and trained and supported by the expert facilitators (PM, AM). Support will include group-based training in evidence-based care strategies for older patients, quality improvement methods, and the i-PARIHS framework; a monthly face-to-face forum for sharing skills and experiences between facilitators; and in-person or telephone support by the expert facilitators as required.

The site facilitator will identify and prepare members of the ward-based multidisciplinary team (MDT, including senior nursing staff, physiotherapist, occupational therapist, dietitian, social worker, speech pathologist, assistant staff, medical staff and others). They will engage MDT members as local champions and informal leaders, both informally and through monthly team meetings. The facilitator will obtain patient feedback through structured interviews and will use a suite of process measures described below to assess current performance. These data will be fed back to the MDT to help them reach consensus on priorities for improvement and to identify potential barriers, enablers and solutions. Solutions might include individual or team actions, or changes to systems or resources. For example, if the baseline data suggests gaps in achieving early mobility, barriers identified by patients and staff might include inadequate pain control, inadequate encouragement, poor team communication of mobility goals, insufficient walking aids, and no walking destination. Solutions in that setting will require consideration of patient assessment, patient and team communication, and ward equipment and design, but may also require support by education and leadership, and multiple interacting strategies may be required to produce measureable improvements. The facilitator will support MDT members using strategies such as marketing, team building, role clarification, audit and feedback, reminders and education. The facilitators will also be encouraged to develop networks, leadership support and monitoring mechanisms at each site, important for sustaining changes [30, 31]. A senior physician designated as the site investigator will support the facilitator and MDT.

Previous successful programs have described an assistant workforce to help ward staff ensure consistent provision of basic care interventions, although the type of assistant varies between models (e.g. trained volunteers [32], nursing assistants [33] or healthcare students [34]). Intervention wards will be provided with a half-time trained multi-professional healthcare assistant, who will undertake self-directed tasks (e.g. providing reading materials, mealtime set-up, reorientation), as well as specific individual or group interventions delegated by health professionals (e.g. assisted mobilisation, exercise programs, encouraging supplement consumption, reminiscence strategies) to support the MDT strategies. The assistant will be recruited locally and undertake training and competency assessment at a central site, with mentoring by the facilitator and MDT, and peer support by monthly teleconference.

### Outcome evaluation

#### Participants and measures

Patients will be eligible to participate if they are aged 65 years or older, and admitted to any study ward during the study period, with an anticipated length of stay of three days or more. Exclusions are patients receiving end of life care, outliers from other teams, patients transferred or discharged from the ward within three days, and those with critical illness or severe cognitive impairment who cannot consent to participate and lack a surrogate able to consent on their behalf. Participants can only be enrolled once in the same study period, but the same participants could potentially be enrolled in both the pre-intervention and post-intervention samples during different hospital admissions.

Data will be collected by trained research assistants from patient (or surrogate) interview at four time points: admission (including assessment of health status prior to admission), day 5 of hospitalisation, discharge and 30 days after discharge from the treating team (the 30-day interview is by telephone). Data items for each interview are shown in Table 2. Additional data from structured review of the medical record will include demographics, diagnosis assigned by the treating team, co-morbidities, number of prescribed medications, previous hospitalisations, admission nursing assessments of common risk factors (vision, hearing, falls, pressure injuries, functional status, cognitive status, continence status), and consultations from allied health professional and other specialties. Baseline data from interview and medical record will be used to construct a frailty index based on the deficit accumulation model [35].

**Table 2:** Interview data collection items and timing. Descriptions and references are included in main text

	Pre-admission	Admission (within72 h)	Day 5	Discharge (within 24 h)	Day 30
ADL	X	X	X	X	X
IADL	X				X
Malnutrition Screening Tool		X			
Depression (PHQ-2)	X				
Self-rated health	X				
Falls	X		X	X	X
Pressure injury		X	X	X	X
Bladder incontinence	X	X	X	X	X
Bowel incontinence	X	X	X	X	X
SPMSQ		X	X	X	X
3D-CAM		X	X	X	
EQ-5D				X	X

ADL activities of daily living: count of basic ADL (bathing, dressing, toileting, transferring, walking across and room, and feeding) requiring human assistance

IADL instrumental activities of daily living: count of instrumental activities (shopping, cooking, housework, transport, using the telephone, managing medications, managing finances) requiring assistance

*PHQ-2* Patient Health Questionnaire-2, *SPMSQ* Short Portable Mental Status Questionnaire, *3D-CAM* 3 min diagnostic assessment for CAM-defined delirium, *EQ 5D* EuroQol health questionnaire

#### Outcomes

The two primary outcomes are any hospital-associated geriatric syndrome and length of hospital stay. Any hospital-associated geriatric syndrome is a composite measure including one or more of delirium, functional decline, falls, pressure injury or new incontinence occurring during the stay under the treating team. These are common, potentially preventable, and associated with poor outcomes [2]. Functional decline is defined as any increase in the number of basic activities of daily living between baseline 2 weeks prior to admission and discharge from the treating team [36]. Delirium is defined by positive 3-min Confusion Assessment Method (3D-CAM) [37] at admission, day 5 or discharge assessment, and/or evidence of delirium in structured medical record screening, as this provides the most sensitive ascertainment of delirium [38]. Falls and pressure injuries are defined as an event reported by the patient at admission, day 5 or discharge assessment, and/or documented evidence in the medical record. New urinary or faecal incontinence is defined as patient-reported incontinence at admission, day 5 or discharge, and/or documented evidence in the medical record, in those participants who reported being continent prior to admission.

Secondary outcomes will include each of the individual geriatric syndromes defined above; discharge destination (home or institutional care); 30-day health status including mortality, readmission, functional status and quality of life; and 6-month readmission and hospital utilisation. Hospital utilisation and readmissions will be obtained from a state-wide database of all public hospital admissions. Quality of life will be assessed using a preference-based utility score (EQ5D) [39] administered at discharge (face-to-face) and 30 days after discharge (telephone) in consenting participants in both groups, enabling estimation of quality adjusted life years gained. Costs to the healthcare system will include implementation costs (project, clinical and management staff time, consumables and overheads) and health services utilisation costs based on length of stay (initial, continuing and readmissions) and costs incurred from adverse events.

Research assistants for each site will be health professionals who are not ward staff and not involved in study design or delivery of interventions. They will undertake a 2-day training course, with a detailed data collection manual of procedures, definitions, and frequently asked questions. They will be supported by site visits by the data manager, weekly teleconference, and telephone and email support throughout the data collection period. Specific training in 3D-CAM delirium assessment will be provided using scored video vignettes provided by the developers and practice scoring of 3 patients under supervision of the chief investigator. Reliability of chart data extraction will be checked by re-abstraction of 5 – 10 charts at each site by the chief investigator and project manager within the first few weeks of project commencement. Data will be entered directly into a secure web-based data collection and management system (RedCAP), with real time data monitoring by the data manager to optimise completeness and integrity of data.

#### Analysis

Participant characteristics will be described and summarised using standard statistics, and compared between sites and between time periods to verify that randomisation achieved reasonable comparability and identify any important changes in patient characteristics over time. Outcome analysis will be at patient level, clustered by ward. Outcomes will be presented as means or proportions and 95% confidence intervals. Analysis of geriatric syndromes will compare the difference in the proportion of participants developing any geriatric syndrome in the pre-intervention and post-intervention samples, under control and intervention conditions, to determine whether there is greater reduction on the intervention wards. Change in average length of stay will be compared using time to event analysis. Participants who are still in hospital at the end of the study period will be censored. Competing outcomes methods will be used to explore the impact of length of stay and discharge destination. Plots of the cumulative risks of discharge by length of stay will be compared between groups to examine the differences in detail and determine whether intervention effect varies by time in hospital. Modelling approaches (such as multiple logistic regression) will be used if needed to adjust for intergroup baseline differences. Planned subgroup analyses will be undertaken by site, by age (<75 versus 75 and older), and by frailty status. Cost-effectiveness will be assessed from the perspective of the healthcare system by modelling the change to total costs and total health benefits, with uncertainties included. The cost per quality adjusted life year gained from wider adoption of the program will be estimated.

We estimate a feasible recruitment of 250 participants for intervention and control ward in each period (i.e. 500 participants each in pre-intervention and post-intervention groups). This provides 80% power to demonstrate 30% reduction in geriatric syndromes in the intervention group, assuming 40% baseline prevalence, and a 15% reduction in acute length of stay assuming baseline of 9 days and SD 7 days. All analyses will be by intention to treat, i.e. all intervention group patients will be analysed as part of the intervention group even if they did not receive specific interventions.

### Process evaluation

Multiple methods will be used to build a comprehensive understanding of how the complex intervention is operationalised in each site [25]. Ward process measures and patient interviews will be fed back by the facilitator to the staff on intervention wards to inform and evaluate improvement strategies. Implementation measures will assess the recipients, context and facilitation activities at each site. System-level data will estimate the effect of improvements at system level.

Ward processes will be assessed by activity mapping, mealtime audits and patient interviews performed on all wards in the pre-implementation and post-implementation periods, and mid-implementation on intervention wards. Mobility and engagement patterns will be assessed using activity mapping for an 8 h day-time period (8 am till 4.30 pm) [40]. In this method of systematic sampling, the auditor observes each patient in a room for a 2 min interval, noting the highest level of a hierarchical list of possible positions (lying, sitting, standing, walking) and activities (sleeping, receiving care, self-care, eating, TV/radio, reading/games/craft, talking, exercising) and in whose company (alone, with visitors, with staff) before moving to the next patient room. Observations are repeated continuously in a consistent order for the 8 h period and summarised as the average proportion of time spent at each level for each patient [40]. Meal-time processes including patient positioning, meal set-up, assistance, interruptions and estimated meal consumption will be measured by auditing one breakfast, one lunch and one dinner on the ward using a structured observation sheet, summarising observations using proportions or averages across all patients [41]. Mealtime audits will include all inpatients present on the ward provided with a meal at the audit time (excluding patients ordered nil by mouth or receiving end of life care).

Patient experience will be assessed by semi-structured 10–15 min interviews administered to inpatients on the study wards aged over 65 who are willing and able to participate in the interview, with a randomised list of bed numbers utilised to select consenting participants until 10 interviews are completed for each ward. Interviews will be analysed using a mixed methods approach, with summary of proportions or averages for quantitative responses and framework analysis of recorded interview transcripts for qualitative responses. Process audits and interviews will be conducted by the ward facilitators pre-intervention (before randomisation occurs) and mid-implementation, and quantitative data will be summarised and fed back to the intervention wards to inform improvement strategies. Post-intervention interviews and audits will be performed on all wards by a trained research assistant not involved in implementation, to reduce the risk of bias.

Implementation measures will include evaluation of context, recipients, facilitation process, and MDT engagement in each intervention ward. Context and recipients will be evaluated by the local facilitator using the i-PARIHS framework [26], assigning scores from −2 (barrier to program goals) to +2 (enabler) for individual domains (e.g. motivation, opinion leaders, networks, senior leadership, learning environment). This iterative evaluation will be supported by reflective discussion with the expert facilitators and other site facilitators, and inform tailored strategies. The facilitation experience will be assessed by interviews by an associate investigator (GH) not otherwise involved in outcome evaluation. Interviews will be undertaken at three time points (early, mid and late implementation) with the 4 site facilitators and the expert facilitators. Open-ended interviews will be recorded, transcribed and analysed thematically, providing transcripts to the participants for checking following completion of interviews. Engagement of the team will be evaluated by meeting attendance (MDT meeting minutes), reflective facilitator feedback (meeting minutes and journal), strategy uptake (meeting minutes and facilitator feedback) and multidisciplinary staff surveys using the NoMAD tool [42].

System-level outcome data will be routinely collected data on acute length of stay, discharge destination, falls and pressure injuries. It will be collected on all 8 wards from January 2015-March 2016 (pre-intervention) and April 2016-June 2017 (post-intervention), displayed graphically and analysed using interrupted time series methods.

## Discussion

There is strong evidence that hospital-acquired geriatric syndromes can be prevented in older inpatients by strategies which engage multiple MDT members [12, 13], but there are still few programmatic approaches to implement these strategies outside of specialist geriatric wards [43]. Eat Walk Engage adapts many principles from the established Hospital Elder Life Program (HELP) [32, 44], including patient-centred care, a strong preventive focus, and interdisciplinary collaboration. Eat Walk Engage also uses strong principles from the emerging field of implementation science to optimise implementation success and sustainability. The i-PARIHS implementation framework, with a key facilitator role and evidence-based quality improvement methods, guides iterative intervention strategies, providing a flexible program tailored to the ward context. Design and staffing of the program have been shaped by local health system features including limited advanced geriatric nursing programs, a strong MDT focus, limited volunteer infrastructure and experience, and an emerging allied health assistant workforce.

As a complex system level intervention, there are several evaluation challenges which must be acknowledged as potential weaknesses. Clinical staff and patients are likely to be aware of the program on the intervention wards, and data collectors may also notice differences between the wards in the post-intervention period, which may introduce bias. This is partly mitigated by using a suite of measures at multiple levels (patient, ward, system), and by training and monitoring of research assistants. Contamination may occur if clinical staff work on both wards and adopt new practices based on their involvement on the intervention ward. However, this is only likely to affect a small number of team members (nursing staff and most allied health professionals do not work across multiple wards), so the impact is likely to be small. Some participants may potentially be enrolled in both the pre-intervention and post-intervention samples if they are readmitted 12 months after first enrolment, which challenges assumptions of independent samples. Variation in patient and disease characteristics between the wards may result in confounding. The relatively small number of clusters is a practical constraint of introducing a complex intervention. Although the program goals, facilitator training and quality improvement approach are consistent across sites, the complex and iterative nature of the intervention means that intervention wards are likely to be at different stages of maturity at the time of evaluation. In a complex and non-standardised intervention, attribution of any impact to specific interventions or resources is difficult, and replication may be challenging.

The study design also offers several strengths in studying a complex intervention. The flexible and tailored nature of the Eat Walk Engage program enables application across a range of hospital and ward types that enhances generalisability of the findings. The prospective use of an implementation framework and a range of process measures will provide rich analysis of factors associated with implementation success or failure, informing further spread of the program if it is successful. Use of a composite geriatric syndromes outcome will expand the evidence for multi-component interventions from the patient perspective. Using wards within the same hospital as concurrent controls will minimise confounding by other health system changes. Training and support for facilitators and assistants and process measures support implementation fidelity.

In summary, this pragmatic study will provide real-world evidence of effectiveness of a novel multi-component program for reducing geriatric syndromes. It will provide evidence of cost-effectiveness and contextual sensitivity for decision makers, as well as detailed implementation data (resources, training and measures) for implementers. It will also provide detailed description of the characteristics, processes and outcomes of care for older patients across a broad range of clinical wards to inform local improvements and priorities.

## Abbreviations

3D-CAM: 3 minute diagnostic interview for confusion assessment method-defined delirium
HELP: Hospital elder life program
i-PARIHS: Integrated promoting action on research implementation in health services framework
MDT: Multidisciplinary team
